# Application of Machine Learning in Epileptic Seizure Detection

**DOI:** 10.3390/diagnostics12112879

**Published:** 2022-11-21

**Authors:** Ly V. Tran, Hieu M. Tran, Tuan M. Le, Tri T. M. Huynh, Hung T. Tran, Son V. T. Dao

**Affiliations:** 1 School of Industrial Engineering and Management, International University, Vietnam National University, Ho Chi Minh City 700000, Vietnam; 2 School of Electrical Engineering, International University, Vietnam National University, Ho Chi Minh City 700000, Vietnam; 3 School of Science, Engineering & Technology, RMIT University Vietnam, Ho Chi Minh City 700000, Vietnam

**Keywords:** EEG classification, seizure detection, machine learning, discrete wavelet transform, binary particle swarm optimization

## Abstract

Epileptic seizure is a neurological condition caused by short and unexpectedly occurring electrical disruptions in the brain. It is estimated that roughly 60 million individuals worldwide have had an epileptic seizure. Experiencing an epileptic seizure can have serious consequences for the patient. Automatic seizure detection on electroencephalogram (EEG) recordings is essential due to the irregular and unpredictable nature of seizures. By thoroughly analyzing EEG records, neurophysiologists can discover important information and patterns, and proper and timely treatments can be provided for the patients. This research presents a novel machine learning-based approach for detecting epileptic seizures in EEG signals. A public EEG dataset from the University of Bonn was used to validate the approach. Meaningful statistical features were extracted from the original data using discrete wavelet transform analysis, then the relevant features were selected using feature selection based on the binary particle swarm optimizer. This facilitated the reduction of 75% data dimensionality and 47% computational time, which eventually sped up the classification process. After having been selected, relevant features were used to train different machine learning models, then hyperparameter optimization was utilized to further enhance the models’ performance. The results achieved up to 98.4% accuracy and showed that the proposed method was very effective and practical in detecting seizure presence in EEG signals. In clinical applications, this method could help relieve the suffering of epilepsy patients and alleviate the workload of neurologists.

## 1. Introduction

Approximately 60 million people worldwide have experienced epileptic seizure [1], which is a neurological disorder represented by brief and unpredictably occurring electrical disturbances in the brain [2]. In neurology, epilepsy is defined as a collection of neurological dysfunctions with a permanent predisposition, which results in recurrent seizures [3]. Symptoms of an epileptic seizure can include mind degradation, cognitive disorders, and frequent body convulsions, consequently worsening quality of life and increasing the number of safety issues. Accurate and early recognition of epileptic seizures is imperative to administer antiepileptic drug treatment to patients and reduce the risk of impending seizures [4]. The usual brain activity of epilepsy patients is classified into four states by analyzing electroencephalogram (EEG) signals. Those four states are [5]: pre-ictal state, ictal state, inter-ictal state, and post-ictal state. An epileptic seizure can cause severe impacts on the patient, such as consciousness deterioration and frequent random body convulsions. By adequately examining electroencephalogram (EEG) signals, a recording of the brain’s electrical activity using non-invasive electrodes placed on the scalp, neurophysiologists can analyze the brain’s neural activities during seizure and nonseizure periods, thus providing timely predictions of upcoming seizures. Researchers have widely studied the advanced recognition of epileptic seizures by using machine learning (ML) models based on EEG signals. To enhance prediction accuracy, numerous publications and methods have been developed, contributing to the precise detection and proper treatment of epileptic seizures and other diseases [6,7].

Seizure event detection indicates the ability to identify the occurrence of seizure events that are happening or have happened in EEG signal patterns. Normally, to classify EEG abnormalities, direct visual examination is initiated by neurologists. However, the process of manually labeling EGG signals is time-consuming and prone to unsatisfactory results [8]. Machine learning can significantly reduce the cost and time of epileptic seizure and EEG signal analysis, as well as the workload of doctors, while also improving diagnosis efficiency. This study primarily focuses on the detection of seizure events in single-channel EEG records with a high degree of accuracy. Multi-channel EEG signals analysis is more common in practical clinical circumstances. However, due to the characteristics of the dataset used, the scope of this work only involves seizure detection from single-channel EEG recordings.

Along with seizure event detection, seizure onset detection is also an imperative part of the automatic diagnosis of an epileptic seizure. Seizure onset detection refers to the ability to precisely predict the beginning of seizure onset before it occurs with the shortest delay [9]. By accurately raising the alarm for seizure onset, proper and timely treatment can be given to patients. Nevertheless, the proposed work suffered from the limitation of not being able to detect the onset of a seizure, as it was not within the scope of our research. This research proposes a model based on machine learning to classify EEG signals into non-seizure and seizure events, and to detect the presence of seizure events in EEG records. The method was tested on the public EEG dataset of the University of Bonn. With the proposed method, the dimensionality of the data is significantly narrowed down, with the results showing significant improvement in terms of performance and computation cost when only the relevant features are used to train the classifiers. This research also aims to maximize the accuracy and performance of the machine learning mode and minimize computational costs. Furthermore, our proposed method has the potential to reduce the heavy clinical workload of neurologists in the medical system and would enable early seizure diagnosis and treatment for patients.

## 2. Literature Review

### 2.1. Related Works

Among the studies, Sharma et al. [1] proposed a novel approach of using analytic time frequency flexible wavelet transform (ATFFWT) to decompose EEG signals, then fractal dimension (FD) was applied to extract the important features. Features were then reduced and ranked to be selected using the student’s *t*-test. After that, selected features were classified using the least squares-support vector machine (LS-SVM), and they achieved the highest accuracy of 100% for the classification of normal and ictal signals, and 99.20% for the discrimination of ictal and non-ictal signals. Additionally, for the implementation of the SVM classifier, Siuly et al. [10] also presented a clustering technique based on LS-SVM for binary classification of EEG signals. The applied technique achieved an average accuracy of 94.18%. Savadkoohi et al. [11] developed a feature engineering-based method in their study to detect meaningful patterns from EEG; they extracted features of EEG signals in time and frequency domains. Afterward, the *t*-test and sequential forward floating selection (SFFS) were applied to select the most optimal features to classify using SVM and KNN; their experiments revealed that SVM slightly outperformed kNN in terms of maximum accuracy in each domain.

Authors from [12] proposed a binary classification framework based on the EEG analysis using discrete wavelet transform (DWT), combined with two classifiers, naïve Bayes (NB) and k-nearest neighbor (kNN), with statistical features extracted from DWT coefficients. They concluded that the NB classifier performed better in most cases. In addition, the computation time of the NB classifier was shorter than k-NN but still provided better accuracy. For the ABCD vs. E case, kNN achieved better results with an accuracy of 97.1%. Other researchers have employed hybrid methods; for instance, Subasi et al. [13] established a hybrid model with genetic algorithm (GA) and particle swarm optimization (PSO) to determine the suitable parameters for the support vector machine (SVM) classifier. It was concluded that the PSO-SVM performed moderately better than GA-SVM, with the percentages of classification accuracy AT 99.38 and 98.75%, respectively. From another previous work by the same group of authors [14], they used discrete wavelet transform to decompose the signals into time-frequency attributes, then the statistical features were extracted from the sub-bands. Principal component analysis (PCA), independent component analysis (ICA), and linear discriminant analysis (LDA) were used to reduce the dataset dimension. In the last stage, the SVM classifier was applied, and it yielded an accuracy of 98.75, 99.5, and 100% for PCA, ICA, and LDA, respectively.

Apart from using the SVM classifier and its variants, several researchers have combined other well-performing classifiers with different feature engineering methods to achieve pre-eminent outcomes. The authors in [15] used line length feature extraction based on wavelet transform multiresolution decomposition, then the desired features were classified using an artificial neural network (ANN). Their paper concluded that the ANN algorithm produced high accuracy with impressive computational performance, provided that the classifier was executed on powerful hardware. Likewise, Tzallas et al. [16] employed a method of analyzing the time-frequency domain, combined with ANN to differentiate the existence and non-existence of seizures. They also attained very promising overall accuracy, ranging from 97.72 to 100% for different cases. The random forest (RF) classifier has also been implemented in some studies, Mursalin et al. [17] presented a novel analysis method for detecting epileptic seizures by using an improved correlation-based feature selection (ICFS) with random forest classifier; the results demonstrated that their method delivered better performance in comparison to other state-of-the-art methods. Other notable methods that coordinate RF classifiers include using iterative filtering (IF) by Sharma et al. [18] and grid search optimization by Wang et al. [19]. These studies concluded that random forest is a reliable and sophisticated classifier when applied in those cases.

Among the aforementioned literature, a paper published by A. Sharmila et al. [12] became the primary reference for this research. In their work on seizure detection, by using DWT to decompose the signal into four sub-bands (coefficients) and extract the statistical features from each of the sub-bands, the results they achieved were satisfactory for different set combinations. However, they only used a one-way ANOVA test to identify feature importance, and the less relevant (redundant) features were not fully assessed and reduced. they also did not take into consideration the problem of overfitting and poor generalization. In supervised machine learning models, overfitting is a major issue. This occurs when a model has been overtrained on training data and is unable to generalize (poor generalization), producing inaccurate results when given new data (testing set), and thus, making the model impractical. This study aims to implement the feature selection technique and use hyperparameter optimization (HPO) to classify EEG data with performance improvement and data dimension reduction. For this research, a combination of ABCD-E was used for classification, as it is said to be close to clinical applications [15]. The problem becomes a binary classification problem with two classes: non-seizure (A, B, C, D) and seizure (E). This problem can also be expressed as differentiating between seizure and non-seizure events in EEG records.

### 2.2. Feature Selection

Feature selection is a method for choosing a subset of important features that can accurately represent data properties, while limiting the impact of redundant or irrelevant features, hence increasing machine learning performance [20,21,22]. For labeled data, the most commonly used feature selection model is the supervised model, which recognizes relevant features that perform best in achieving the objective of the supervised model, such as classification. Generally, a supervised model can either be a filter or wrapper method. An additional approach deriving from the two previous ones was called a hybrid method [23,24,25].

**Figure 1:**
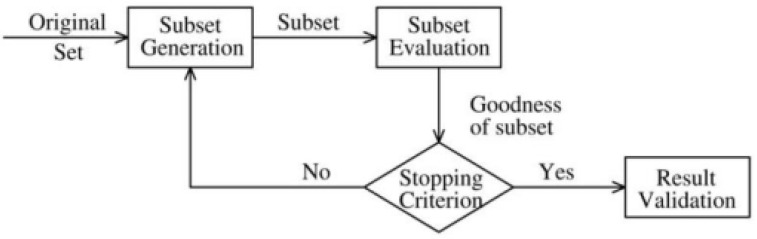
Feature selection flowchart.

A general feature selection process [26,27,28,29] is depicted in Figure 1, which includes four steps: subset generation, subset evaluation, stopping criterion, and result validation. Subset generation uses a specific search strategy to generate feature subsets. Then, each subset is assessed using a specific evaluation criterion and compared with the prior best candidate subset. The subset generation and evaluation process are repeated until a specified stopping criterion is met. Some possible stopping criteria include when the search is finished, a predefined limit is reached, the result does not change after a specified time (or number of iterations), etc. Finally, prior knowledge or test data is used to validate the best feature subset.

For the feature selection process in this work, binary particle swarm optimization (BPSO) was chosen to be the heuristic search algorithm, and naïve Bayes was selected to be the induction classifier because of its fast computation capability that is suitable for a wrapper method [30].

### 2.3. Hyperparameter Optimization

Tuning hyperparameters (HPs) is the process of determining the ideal combination of hyperparameters that enhances model performance; it is an important stage in developing an effective machine learning model, as hyperparameters dictate how the model is structured preliminary to the training phase. Hyperparameters are used to either configure the ML model (e.g., the penalty parameter C in SVM) or to indicate the algorithm used to maximize the performance of the model (e.g., the kernel type in SVM) [31].

HPO is widely applied because it possesses many benefits, as follows [32]: it saves time needed for tuning the hyperparameters and reduces the human effort required, it improves the performance of the ML model, and improves the reproducibility and fairness of the models. To identify ideal hyperparameters, it is important to use the right optimization technique. Two popular techniques are grid search and random search. 

Grid search (GS) is an exhaustive search that evaluates all hyperparameter combinations given to the grid of configurations. If sufficient resources are provided, GS can lead to the most accurate results. GS is appropriate for a variety of hyperparameters with a small search space [33]. The idea of GS is to evaluate the Cartesian product of a user-specified finite set of values. 

**Figure 2:**
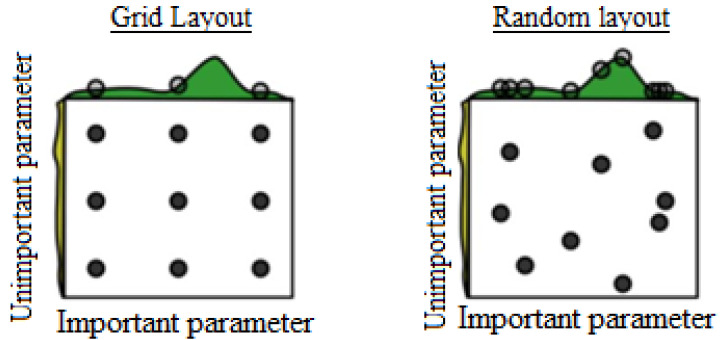
Grid search and random search comparison.

Random search (RS), or known as randomized search, is a similar but improved version of grid search. When the predefined budget is depleted or target accuracy is achieved, the search procedure ends. Similar to GS, RS is a computationally intensive method. However, in many cases, RS is proven to be more effective and produce better outcomes than GS [31,33]. The difference between grid search and random search is illustrated in Figure 2 [34], given two parameters: important and unimportant. For GS, a 3 × 3 grid is formed with nine combinations, it only searches three different values for the important parameter in nine iterations. In contrast, RS can search nine different values for the same nine iterations. Thus, it is much easier for RS to search for the important parameters as it explores the space more widely.

To conclude, in most cases, especially in high-dimensional search space, RS is shown to be more effective and efficient compared with GS as it can explore a large search space and has less time complexity. Thus, random search was chosen to be the HPO method in this research.

### 2.4. Classification

#### 2.4.1. Support Vector Machine (SVM)

Support vector machine (SVM) is a popular form of the supervised machine learning algorithm that trains a model and helps it learn by classifying points in the space features. SVM’s function is based on the idea of a margin, which is either side of a hyperplane that separates two data classes. The main principle of SVM is to find the optimal hyperplane for the separation of classes by maximizing the margin of the support vectors [35,36].

#### 2.4.2. K-Nearest Neighbors (KNN)

K-nearest neighbor (kNN) is an instance-based algorithm. It works on the assumption that the instances in a dataset are likely to be found near other instances with similar features. If each instance has a classification label, the label of an unclassified instance can be identified by examining the class labels of its closest neighbors. The kNN algorithm works by locating the *k* nearest instances to the query instance and defining its class by identifying the single most recurrent class label [37].

#### 2.4.3. Decision Tree (DT)

Decision trees (DT) classify instances by sorting them based on the feature (attribute) values of the instances [37]. The root node, internal node, branch, and leaf node are the four basic sections of a decision tree. The decision tree starts at the top and gradually moves down, with each internal node representing a feature test, each branch representing the output of a feature test, and each leaf representing the classification classes [38]. The leaf node is the node that cannot be split further; hence, it does not produce a child node.

#### 2.4.4. Random Forest (RF)

Random forest consists of a combination of decision trees made from the random selection of samples of the training data. Random features are selected in the induction process. Predictions are proceeded by aggregating the predictions of the ensemble with the most votes. Each tree is grown to the maximum possible extent, and no pruning is used [39,40]. 

## 3. Methodology

**Figure 3:**
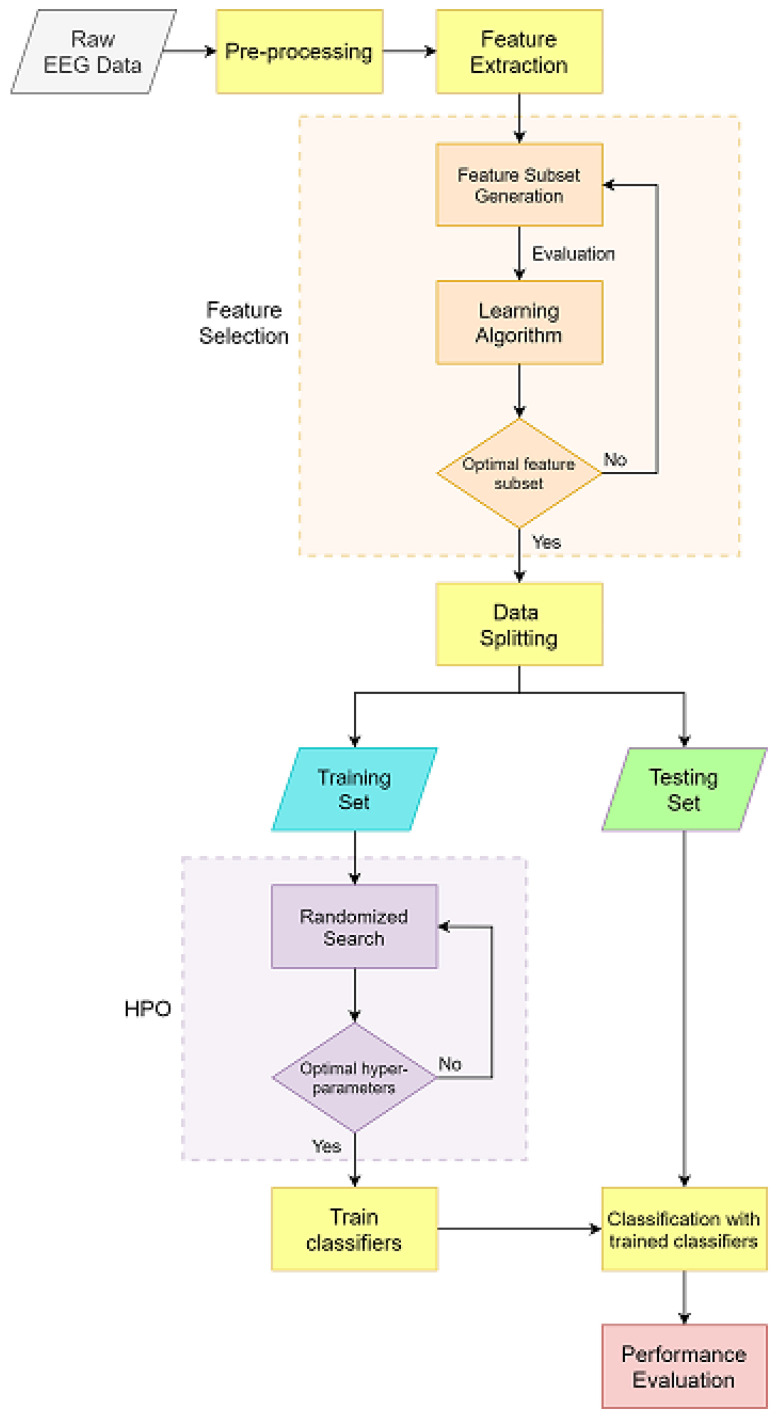
Flowchart of the proposed approach.

All steps of our approach are depicted in Figure 3. The raw signal data is first converted into a 2D table format. As raw data cannot be used to provide useful information, this is done to make analysis easier and more accessible. This step also makes the dataset supervised, allowing the class attributes to have a range of possible values. In raw biological signals, noise and artifacts often exist due to muscle and eye movements. After converting raw data to a 2D table, these artifacts must be filtered to reduce their impact on feature extraction. Feature extraction is performed after the EEG signal has been pre-processed. If the raw EEG dataset is directly applied to a machine learning classifier, the classifiers cannot obtain enough useful patterns and result in poor performance. Thus, feature extraction is an essential stage to capture informative features and obtain useful information from the raw EEG dataset. After extracting the features, it is a common issue that not all features are truly relevant or contribute to the efficiency of the model. In addition, it can also cause dimension redundancy. To achieve maximum classification accuracy with minimal computational effort, it is crucial to select the most relevant feature subset from the original feature set, and that subset should be most suitable for achieving good results in the classification task [23]. Therefore, feature selection is used to select a subset of highly informative features, as well as to remove the irrelevant ones, and those selected relevant features will be used in the subsequent steps. After the preceding steps, the data (feature subset) is divided into training and testing datasets. Classification between seizure and non-seizure EEG records is carried out using machine learning classifiers (classification models). The training dataset will be used to train the classifier in learning the pattern and calculating the optimal way to assign class labels to the input samples (data). Consequently, the performance of a trained classifier will be tested using the testing set. This also helps to validate whether the classifiers are able to predict the pattern of the new, unlabeled data. In ML models, hyperparameters should be adjusted beforehand, as they have an impact on the classifier’s performance. Therefore, hyperparameters optimization (HPO) is implemented before training the classifier. The aim of HPO is to find the optimal hyperparameters of a given machine learning algorithm that delivers the best performance. The ‘No Free Lunch’ theorem for supervised machine learning by David H. Wolpert [41] states that no single model works best for every problem. Therefore, four different classifiers, including support vector machine (SVM), k-nearest neighbors (KNN), decision tree (DT), and random forest (RF) methods were applied in this model. 

### 3.1. EEG Dataset from Bonn University

This research used a publicly available dataset provided by Andrezak et al. [42] at the University of Bonn, Germany. The dataset included five sets A, B, C, D, E, and each set contained 100 single-channel EEG segments with a duration of 23.6 s and were digitized at a sampling rate of 173.61 Hz. Therefore, each data segment included 173.61 × 23.6 = 4097 sample (data) points. Moreover, in the original database, band-pass filter settings of 0.53–40 Hz (12 dB/oct) were used.

In the dataset, as illustrated in Table 1, Set A and B included information taken from scalp EEG recordings of five healthy volunteers in an wakeful state, with their eyes open (Set A) and closed (Set B). On the other hand, Sets C, D, and E were extracted from EEG recording archives of presurgical diagnoses from five epileptic patients, thus the EEG signals of these patients were taken intracranially. Information in Set C was recorded from the hippocampal formation of the opposite hemisphere of the brain, whereas those from Set D were obtained from within the epileptogenic zone. Segments of Set C and D contained activity measured during non-seizure intervals (inter-ictal). Only Set E contained signals during seizure activity taken from all recording locations with ictal occurrence.

### 3.2. Model Development

#### 3.2.1. Data Preprocessing

The raw EEG data consisted of 5 sets (Set A–E), with each set containing 100 EEG segments, and each segment having 4097 sample points.

**Figure 4:**
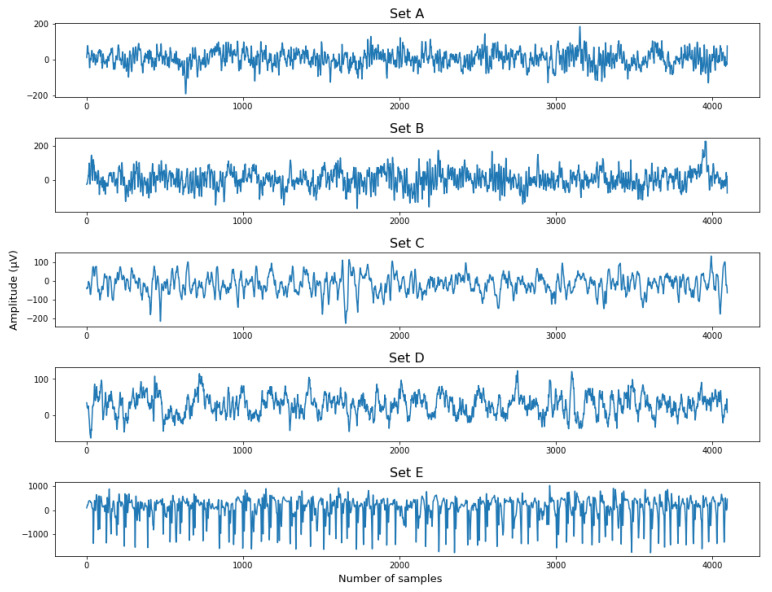
Visualization of EEG signals from Sets (**A**–**E**).

**Figure 5:**
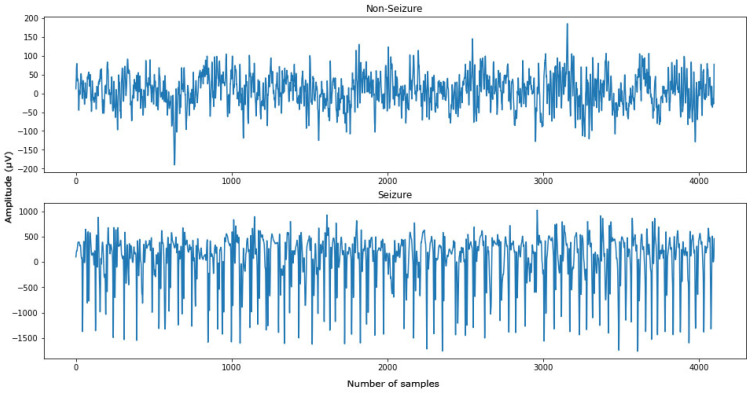
Visualization of ‘non-seizure’ and ‘seizure’ EEG signal.

The visualization of the EEG signal of each set is shown in Figure 4. As the classification problem was between Sets ABCD and Set E, segments from Sets A–D were merged together as ‘non-seizure’ segments, while segments from Set E were ‘seizure’ segments. Figure 5 illustrates the EEG signal after being categorized into ‘non-seizure’ and ‘seizure’ classes.

**Figure 6:**
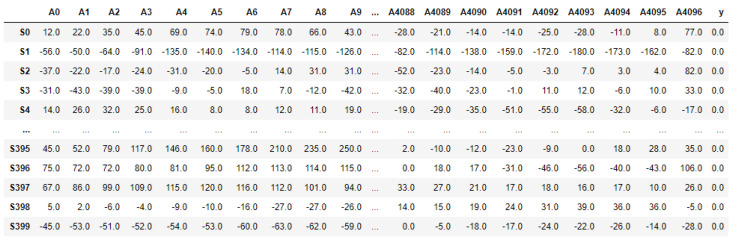
Table view of the first 400 segments.

**Figure 7:**
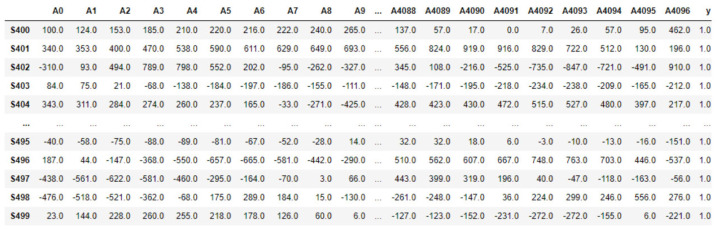
Table view of the last 400 segments.

After loading into Python, the raw dataset was transformed into a 2D table, with a total of 500 segments denoted as *Si* with *i* ∈ [0, 499] in a row, and each sample point in the segments denoted as *Aj* with *j* ∈ [0, 4096] in the column. The last column ‘y’ was the label of each segment. Figure 6 shows segments S0 to S399 with label 0, for ‘non-seizure’, and Figure 7 shows segments S400 to S499 with label 1, for ‘seizure’.

Wavelet transform (WT) is a technique based on multi-resolution (time-frequency) analysis. WT can effectively provide precise information at both low-frequencies and high-frequencies as EEG signals contain low-frequency information with a long period and high-frequency information with a short period [15]. Wavelet transform comes in two distinct forms: continuous wavelet transform (CWT) and discrete wavelet transform (DWT). DWT is often preferrable because it can act as a filter bank to decompose the signals into different sub-bands and remove noises in the signals. With a given wavelet function ψ (*t*) that is scale-shifted by two parameters: *a_j_* = $2^{j}$ (scaling parameter) and *b_j,k_* = $2^{j}k$ (translation parameter), DWT of a signal x(t) can be formulated as follows [17]:

(1) $$d_{j,k}=\frac{1}{\sqrt{\left|2^{j}\right|}}\int_{-\infty }^{\infty }x\left(t\right)\psi \left(\frac{t-2^{j}k}{2^{j}}\right)dt\;$$

where $d_{j,k}$ is the wavelet coefficients, *k* represents the location, and *j* represents the level of decomposition. There are many types of wavelet functions with different orders. However, Daubechies wavelet of order 4 (db4) was chosen because it was shown to be suitable for detecting changes in EEG signals [14,15].

In the first stage of the DWT, the signal x[n] goes into a filter bank, which consists of high-pass h[n] and low-pass g[n] filters. The outputs that come from the first low-pass and high-pass filters are described as 1st level approximation (A1) and detailed (D1) coefficients (sub-bands), respectively. For the 2nd decomposition level, the low-pass coefficient (A1) is iteratively filtered by the same technique to produce coefficients A2 and D2; the process stops when the maximum or desired level is reached. At each level of decomposition, the samples of output signals (with half the frequencies of the original signal) are reduced by a factor of two, according to Nyquist’s rule.

**Figure 8:**
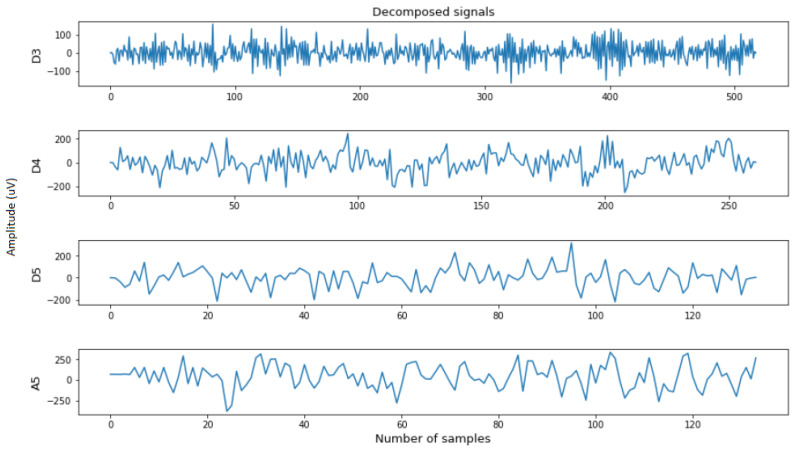
Visualization of D3, D4, D5, and A5 coefficients.

In this research, the selected decomposition level was 5 as shown in Figure 8, and the original signal was decomposed into five detail coefficients (D1, D2, D3, D4, D5) and one final approximation coefficient (A5). Furthermore, only coefficients from the 3rd to 5th level (D3, D4, D5, A5) were chosen to extract the features, as they were shown to be efficient in providing meaningful characteristics from the sub-bands [12,13,14].

#### 3.2.2. Feature Extraction

The performance of classification problems mainly depends on the extracted features. To achieve satisfactory classification results, distinctive features are required to be extracted. Thus, statistical features of wavelet coefficients were extracted from each of the four sub-bands; therefore, the total number of features was 10 features × 4 sub-bands = 40 features. Assuming the sample values of a signal were represented as *X = X_1_*, *X_2_*, *X_3_*, …, *Xn*, with *n* as the maximum sample length, then the features derived from the coefficients in each sub-band were the minimum, maximum, number of zero-crossings, mean, median, variance, standard deviation, root mean square, skewness, and kurtosis.

After extracting features from each coefficient, the total features obtained was 40. Each feature was denoted as *fi*, with *i* ∈ [1, 40]. Table 2 shows the summary of features extracted from each coefficient.

**Figure 9:**
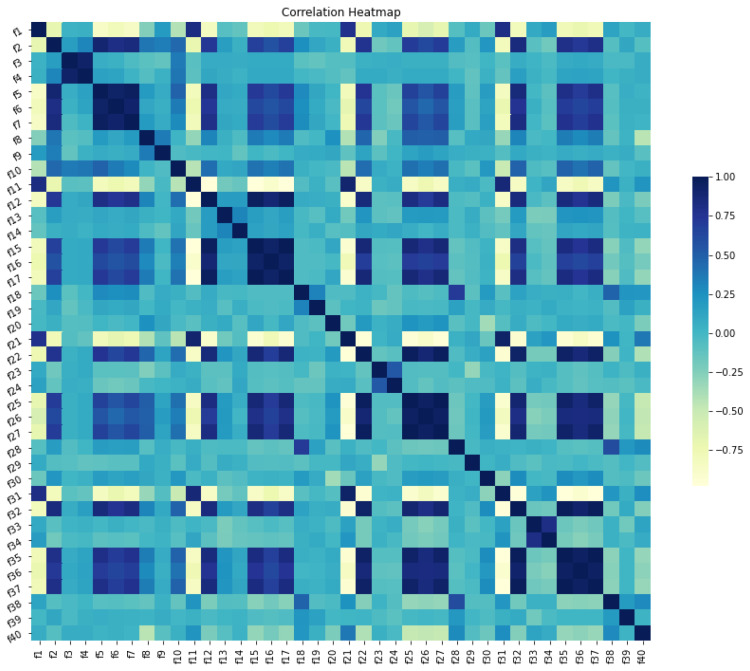
Visualization of D3, D4, D5, and A5 coefficients.

As can be seen from the heat map in Figure 9, standard deviation, variance, and root mean square have the highest correlation with each other and the lowest correlation with the minimum. Additionally, the median and mean are also highly correlated with one another. Strongly correlated features should be diminished, as they do not contribute to the improvement of the model’s performance. Removing them can also reduce the data dimensionality and speed up the computing process.

The feature data with a shape of (500, 40) was split into train data and test data, with a ratio of 75/25. Hence, the data size of the training set was (375, 40) and that of the testing set size was (125, 40).

#### 3.2.3. Baseline Results

In the baseline run, feature selection and HPO were not performed. All 40 features were classified by the four classifier models with their default hyperparameters (HPs). After the classifiers had been trained using the training set, their efficiencies were validated with the testing set.

According to Table 3, in the baseline run, random forest outperformed all of the other classifiers on all metrics, and it achieved an accuracy of 96.8%; however, RF also took the longest time to compute as it is an ensemble of many decision trees. In contrast, KNN generally had the lowest score, with an accuracy of 95.2%.

### 3.3. Model Improvement

#### 3.3.1. Feature Selection

In this approach, the wrapper-based feature selection method uses BPSO as the search strategy and a Gaussian naïve Bayes classifier as the predictor. As FS is a binary optimization issue, the solution is represented by a binary vector, with 1 indicating that the relevant feature is selected and 0 indicating otherwise. In addition, the number of features determines the solution size.

Particle swarm optimization (PSO), introduced by [43], is a popular metaheuristic algorithm inspired by the swarming behaviors of some species in nature. In the PSO search strategy, every particle (candidate solution) is a point located in a dimensional search space. Particles have their own memories, which store both their own and the swarm’s best experiences in finding the perfect solution in the search space. Each individual solution traverses the search space at a dynamically modified velocity that is influenced by its own experience as well as that of other particles. In the first stage, the initial number of particles in the swarm is distributed at random over the search space. Each particle’s position is represented by a vector, where D is the search space’s dimensionality. The velocity of the search ***v_i_*** = (***v*_*i*1_**, ***v*_12_**, …, ***v_id_***) increases as each particle with coordinates ***x_i_*** = (***x_i_*_1_**, ***x_i_*_2_**, …, ***x_id_***) travels in the search space to locate the best solution. During the movement, particles adjust their locations and velocity based on their own and neighbors’ experiences. Each particle has a memory that stores the place where it had its best experience, which is represented as P_best_. The best experience of the whole particle swarm is called the global best, denoted as G_best_. The position and velocity of each particle updated in each iteration are formulated according to Equations (2) and (3) [44].

(2) $$x_{id}\left(t+1\right)=x_{id}\left(t\right)+v_{id}\left(t+1\right)\;$$

(3) $$v_{id}\left(t+1\right)=w*v_{id}\left(t\right)+c_{1}*r_{1}*\left(P_{id}-x_{id}\left(t\right)\right)+c_{2}*r_{2}*\left(P_{gd}-x_{id}\left(t\right)\right)\;$$

where t is the iteration in the process of evolution, d ∈ D is the *d*th dimension in the search space, w is the inertia weight that controls the effect of prior velocities on the current one, and ***c*_1_** and ***c*_2_** are cognitive and social acceleration coefficients, respectively. These two parameters represent the weighting of stochastic acceleration. ***r*_1_** and ***r*_2_** are two randomly and uniformly distributed numbers. ***P_id_*** also is P_best_, representing the local best in the *d*th dimension, and g_best_ represents the global best in the dth dimension.

The search algorithm stops when a predefined stopping criterion is satisfied. In this study, the stopping criterion was when the maximum number of predefined iterations is reached. However, feature selection and many optimization problems occur in discrete search spaces [44]. Due to this reason, authors in [45] introduced a discrete binary version of PSO (BPSO) that could solve optimization cases in discrete domains. In BPSO, the update rule for the velocity remains the same as the original PSO, the difference is that variables ***x_id_***, ***P_id_***, and P_gd_ can only hold binary values, 0 or 1. As a result, the velocity will represent the probability of a particle in the position vector having value 1. In BPSO, the particle’s current position is updated according to Equation (4), using the probability value T (*Vt*) obtained from Equation (5).

(4) $$x\left(t+1\right)=\{\begin{array}{c} 1\;if\;rand<S\left(v\left(t+1\right)\right)\\ 0\;otherwise \end{array}\;$$

(5) $$S\left(v\left(t+1\right)\right)=\frac{1}{1+e^{-v\left(t\right)}}\;$$

where rand is a random number in the range [0, 1], and S(v(t+1)) is the sigmoid function.

A naïve Bayesian classifier works based on the Bayesian rule and probability theorems. It uses the assumption that the attributes are conditionally independent on the class label given [46]. A fitness function is deployed to measure the quality of optimizer solutions and guide the wrapper algorithm. The objective is to maximize the model performance and minimize the feature space; the fitness function inspired by the work [47] was used:

(6) $$fitness=\alpha *E_{R}+\beta *\frac{N_{S}}{N_{f}}\;$$

where ***α*** ∈ [0, 1] and ***β*** = 1 − ***α*** indicates the importance (trade-off) between the error rate of the classification performance, which equals ***E_R_*** =1 − Accuracy, and the size of feature subset ***Ns*** regarding the total number of features ***N_f_***. In this study, the value ***α*** = 0.99 was also adopted from [47]. The maximum iteration that feature selection with BPSO (FS-BPSO) will process is 1000 iterations. As the goal was to find the global-best solution, the number of initial particles was set to be equal to the number of neighbors that the particle considered. The BPSO parameters were arbitrarily configured.

- Cognitive coefficient ***c*_1_**: 0.7
- Social coefficient ***c*_2_**: 0.7
- Inertia weight ***w***: 0.5
- Number of particles: 40
- Number of neighbors that the particle considers *k*: 40

The optimal fitness value obtained from FS-BPSO was 0.0104; only 10 relevant features are selected out of the total original 40 features by FS-BSPO, thus reducing the feature dimension by 75%. The selected and unselected features are shown in Table 4.

**Figure 10:**
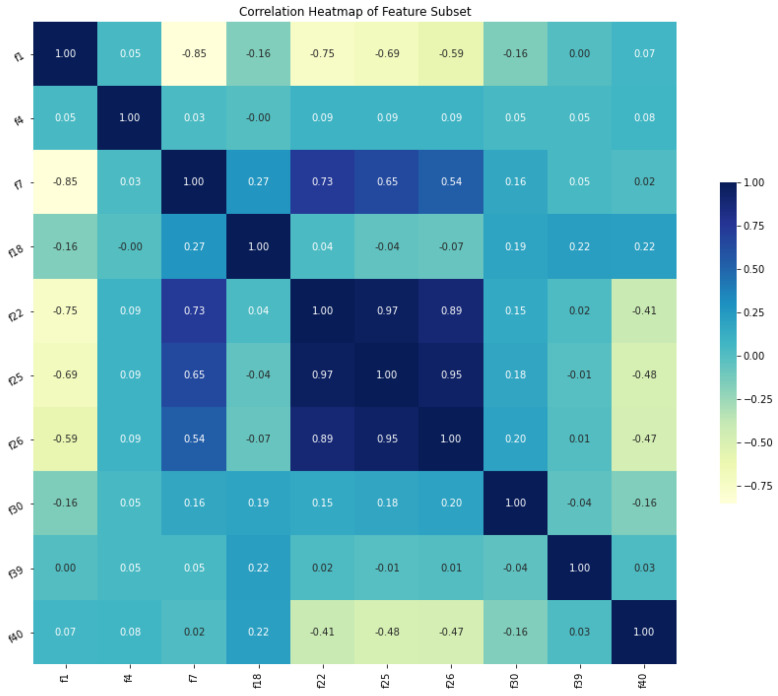
Correlation heat map of feature subset.

Figure 10 is the correlation heatmap of the feature subset consisting of 10 selected features. Only features ‘f22’, ‘f25’, and ‘f26’ exhibited a strong correlation with each other; conversely, the remaining features shared a weak to almost no correlation between them.

#### 3.3.2. Hyperparameter Optimization

As random search was chosen to be the search method for HPO, RS was run for 20 iterations on the training set for each classifier. The hyperparameter search space of each model is provided in Table 5.

## 4. Experimental Results

### 4.1. Performance Evaluation

The efficiency of the classifiers was validated by some performance metrics, such as the confusion matrix, accuracy, precision, recall, F1-score (F-measure), and AUC-ROC curve. There were four possible classification outcomes as shown in Table 6:

- True Positive (**TP**): the model predicted positive, and the actual value is positive (true). Real-world interpretation: there is a ‘seizure’ event in the EEG record, and the classifier accurately detected that EEG record as a ‘seizure’ case.
- True Negative (**TN**): the model predicted negative, and the actual value is negative (true). Real-world interpretation: there is no presence of ‘seizure’ in the EEG record (normal EEG record), and the classifier correctly detected that EEG record as a ‘non-seizure’ case.
- False Positive (**FP**): the model predicted positive, and the actual value is negative (false/Type 1 error). Real-world interpretation: there is no ‘seizure’ occurrence in EEG signal (normal EEG signal), but the classifier detects that signal as a ‘seizure’ case, thus the classifier detected inaccurately.
- False Negative (**FN**): the model predicted negative, and the actual value is positive (false/Type 2 error). Real-world interpretation: the classifier detects the EEG recording that has ‘seizure’ as a ‘normal’ case, thus the classifier detected incorrectly.

**Accuracy**: the ratio between the correct predictions and total number of instances. This is a commonly used metric and will be used to compare with the results from the key reference.

(7) $$Accuracy=\frac{TP+TN}{TP+TN+FP+FN\;}\;$$

The accuracy has traditionally been the most widely used empirical metric. However, in the context of imbalanced datasets, accuracy alone is not a valid metric because it does not distinguish between the number of correctly categorized cases in various classes. As a result, it may lead to incorrect conclusions and not be able to clearly interpret the results in imbalanced data [48]. Furthermore, from the standpoint of real-world problems, the class with the fewest instances is frequently the class of interest. In imbalanced problems, misclassifications in the minority class will not have a significant impact on accuracy [49]. Therefore, other performance measures that are typically used in imbalanced binary classification problems were also evaluated.

**Precision**: the ratio of true positive predictions with total positive instances. **Precision** describes how well the model predicts the positive class; it also represents the model’s ability to accurately predict positives out of all the positive predictions it has made.

(8) $$Precision=\frac{TP}{TP+FP}\;$$

**Recall**: also called sensitivity or true positive rate is the ratio of true positive instances that are correctly classified. Recall represents how many predictions made by models are actually positive out of all true predictions made.

(9) $$Recall=\frac{TP}{TP+FN}\;$$

**F1**-score: also called F-measure, is the harmonic mean between precision and recall values.

(10) $$F1=2\frac{precision\;*\;recall}{precision+recall}\;$$

AUC-ROC curve: a performance metric for the classification problems at different thresholds and is a useful metric in imbalance problems. 

### 4.2. Proposed Model Results 

The hyperparameters listed in Table 7 were acquired from random search HPO. Random search in this work used accuracy as the scoring metric.

**Figure 11:**
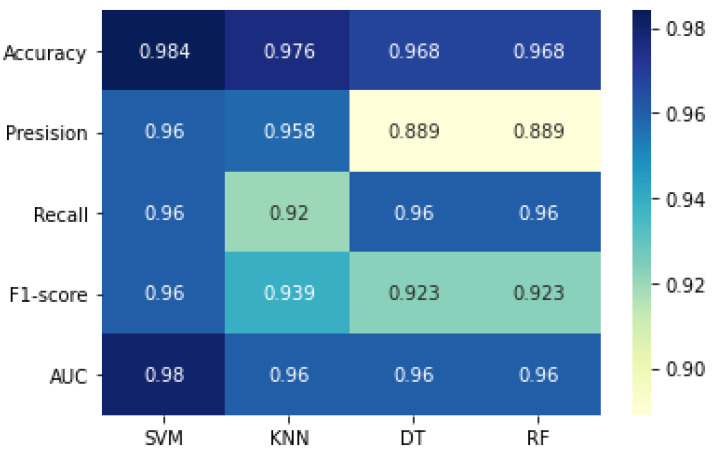
Results heatmap of all classifiers.

**Figure 12:**
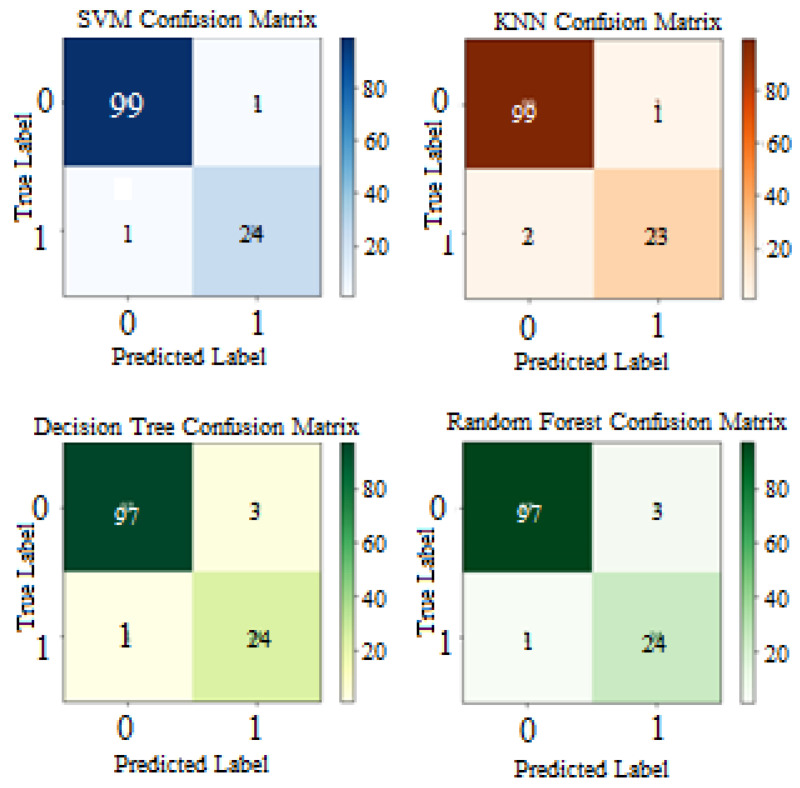
Confusion matrices of all classifiers.

**Figure 13:**
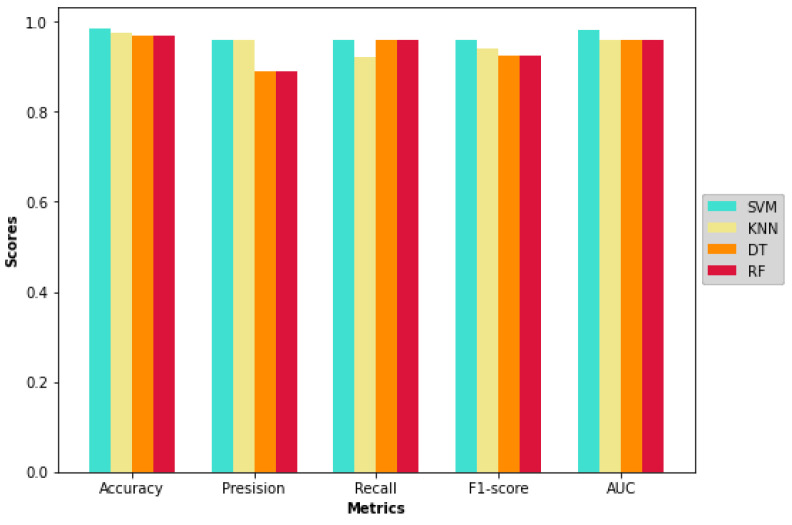
Results comparison of all classifiers.

The classifiers configured with the hyperparameters were used to perform classification on the testing set. The result summary of the proposed model on the first run is indicated in Table 8 and is visualized from Figure 11, Figure 12 and Figure 13. Note that the term “initial results” used from now on indicates the first run results of the proposed model and differs from “baseline results”. As shown in the table and figures, the proposed approach achieved significant improvements compared with the baseline results and results from key references. The SVM classifier outstripped all the other classifiers as it yielded the highest accuracy of 98.4%. The precision, recall, and F1-score of SVM were also overwhelmingly greater than those of the other classifiers.

The runner-up was KNN, which achieved an accuracy of 97.6%. Interestingly, decision tree and random forest produced similar results. To further examine the results, the confusion matrix is shown in Figure 12.

Both SVM and KNN accurately predicted 99 out of the actual 100 ‘non-seizure’ cases. However, SVM performed better in predicting the ‘seizure’ instances, with only 1 instance falsely predicted. It should be noted that in healthcare and clinical applications, false negatives are considered to be more important than false positives. In EEG detection problems, the model can accidentally raise a false alarm when predicting a ‘non-seizure’ case as ‘seizure’, but it should not mistakenly predict ‘seizure’ as a ‘non-seizure’ case. Therefore, instances of false negative should be minimized as much as possible, which means the recall score should be maximized. The acquired results showed that most classifiers yielded a high recall score of 96%, indicating that the model was highly capable of detecting real ‘seizure’ occurrences, allowing timely treatment of ‘seizure’ patients. Meanwhile, precision scores showed that when the model predicted if a segment had a ‘seizure’, it was correct 89–96% of the time.

When observing the F1-score, the classifier that obtained the most harmonic balance between precision and recall was SVM, with a score of 96%.

**Figure 14:**
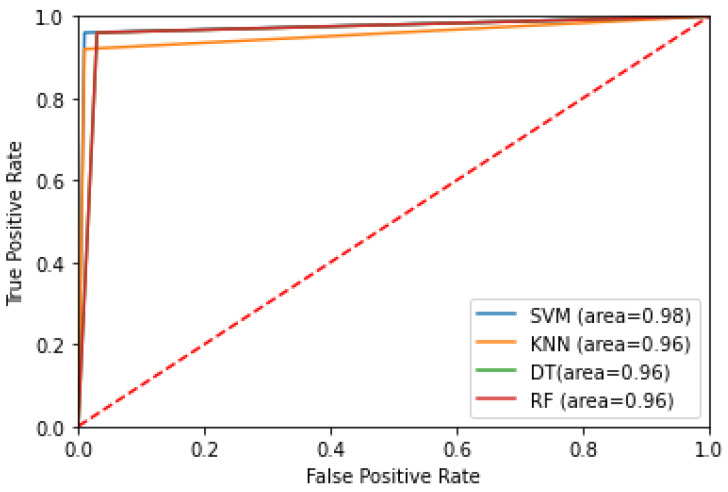
ROC curve of all classifiers.

As shown in the ROC Curve in Figure 14, SVM has the highest AUC value of 0.98, whereas the other classifiers share the same score of 0.96. In general, a value of AUC over 0.9 is measured as significant [50]. The achieved AUC scores also indicate that there is approximately a 96 to 98% chance that the model will correctly distinguish between ‘seizure’ and ‘non-seizure’ occurrences in EEG segments.

### 4.3. Results Comparison

#### 4.3.1. Compare with Baseline Results 

The comparison of rate of change is presented in Table 9, where the results obtained by the proposed model are shown along with the baseline results from the baseline models. 

The efficiency of the proposed approach is demonstrated by the striking improvement in the SVM classifier, as it yielded higher scores in nearly 47 percent less time than the baseline models.

Other classifiers, such as KNN, DT, and RF, also exhibited an improvement in all the metrics, and their running times were also reduced. However, RF suffered a slight drop in AUC score.

The computational time between baseline and proposed models are listed in the Table 10 below:

From Table 10, it can be concluded that our proposed model outperformed the other approaches. The model reduced processing time up to 46.5% for the SVM model. Compared with other baselines for DT, RF, and KNN, our approach was also faster with improvements of 19.3, 18.7, and 11.1%, respectively. 

#### 4.3.2. Compare with Key Reference 

The authors in the key reference used accuracy as the main scoring metric, along with sensitivity (recall) and specificity (equals false positive rate). The results achieved from the proposed model with SVM appear to outperform the results from key references regarding all three metrics and are shown in Table 11.

### 4.4. Analysis 

#### 4.4.1. Model Validation 

**Figure 15:**
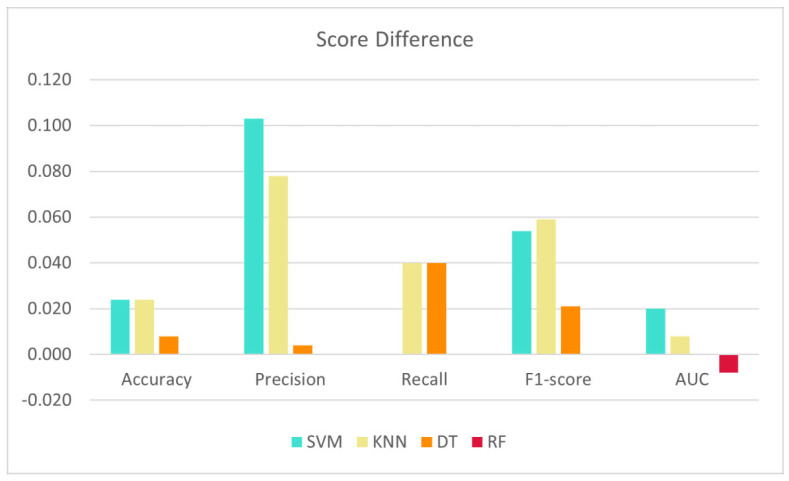
Score difference between the proposed model and baseline results.

Ten trials were performed in this study to validate the model’s stability, with the initial results corresponding to Trial 1. The results of each classifier over ten trials are presented from Table 12, Table 13, Table 14 and Table 15. The standard deviation for each classifier over 10 trials are depicted in Figure 15.

**Figure 16:**
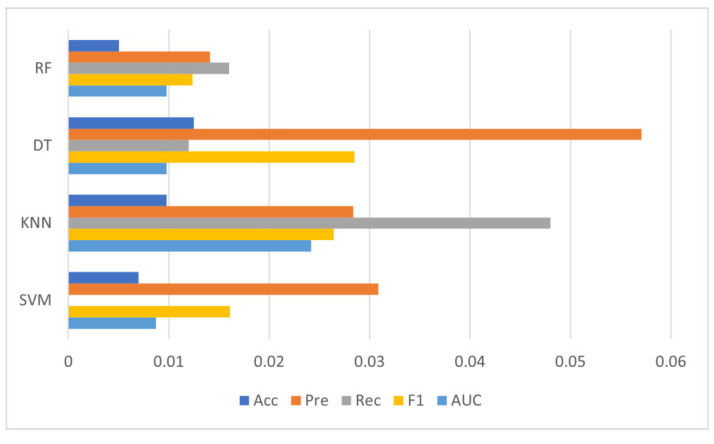
The standard deviation of classifiers over 10 trials.

From Figure 16, it can be concluded that SVM, Decision Tree, and Random Forest classifiers maintained a stable performance throughout 10 trials. Among four classifiers, random forest exhibited the most consistent and steadiest performance on all five metrics, as it had the lowest standard deviation. The performance of SVM also remained comparatively unchanged, especially with the recall score (Std = 0). On the contrary, both decision tree and KNN witnessed considerable variation in their performances over 10 trials. To be specific, recall scores fluctuated the most for KNN, whereas precision scores fluctuated the most for decision tree.

#### 4.4.2. Sensitivity Analysis

Sensitivity analyses were carried out to analyze how sensitive the model’s performance was when changing various factors. In this section, two scenarios will be considered: a change in the number of iterations in BPSO, and a change in both the number of particles and neighbors to be considered in BPSO.

##### Scenario 1: Iteration in FS-BPSO

In the initial run of the proposed framework, the number of iterations used in FS-BPSO was 1000; the different iterations that were tested were 50, 100, 500, 3000, and 5000. Meanwhile, the other parameters remained unchanged.

As shown in Table 16, SVM experienced a substantial rise in all metrics when the number of iterations increased from 50 to 1000. Its performance peaked at the 1000th iteration. However, this is also when diminishing returns would occur, as the performance collapses from the 1000th iteration onward. Similarly, the same thing can be said for KNN. In contrast, only the decision tree produced better performance from the 3000th iteration; random forest remained fairly stable.

##### Scenario 2: Initial Swarm and Considering Neighbors

In the initial setting, the numbers of the initial swarm and neighbors *k* were equally set to 40. In this scenario, various values were set for both the number of particles and neighbors *k*, and, similar to the first scenario, the remaining parameters stayed the same.

Table 17 shows that changing both the number of particles and 52 neighbors led to a steady performance growth for all models, from values 10–40. KNN and SVM exhibited a drop in performance between values 40 and 60.

Notably, both decision tree and random forest remained virtually consistent at different values, with only a slight drop in recall performance from values 40 to 60.

### 4.5. Summary

To summarize our findings, in most circumstances, the SVM classifier outstrips the other classifiers in terms of performance and efficiency. In the first run, it achieved a remarkable accuracy of 98.4% within a short amount of time. KNN also produced promising initial results, with an accuracy of 97.6%. The impressive and reliable performance of SVM and KNN is also shown in [11,51]. However, when it comes to stability, random forest and decision tree have been shown to be more stable than SVM and KNN in different trials and scenarios.

The Table 18 below shows the results comparison between the proposed method and some recent studies on the same dataset, with regard to the highest accuracy in the ABCD-E case.

## 5. Conclusions

In conclusion, this paper proposed a machine learning-based framework in detecting epileptic seizure (ES) events from EEG records. The proposed model successfully extracted insightful features from raw EEG signals based on discrete wavelet transform analysis. DWT also helped remove noises and artifacts found in the original signal, as their presence makes feature extraction very difficult in the subsequent stages. By using feature selection, the dimensionality of the data was significantly reduced by 75% in the first run, and this resulted in a spectacular improvement in terms of performance and computation cost, with about 47% time saved when only the relevant features were used to train the classifiers. Lastly, model validation and sensitivity analysis were carried out to validate the effectiveness and practical implementation of the model. The highest accuracy obtained was 98.4% with the SVM classifier, and the Pre and Rec scores were also satisfactory with a 96% score. In medical applications, the proposed approach does not only have potential to alleviate the substantial clinical workload of the neurologist, but would also allow for early seizure detection and treatment, thereby improving patient health and life quality.

Despite the merits of the proposed model, it still had some pitfalls that are worth addressing, as follows [3]:

- Reproducibility: although the model has attained desirable results in the prototype, it struggles from deficient reproducibility when used in clinical practices. This is because ES prediction is a multiscale problem that is heavily influenced by the patient’s profile.
- Generalization: another drawback is the lack of generalizability of this ES detection model among various seizure types and patients. Seizures often vary between patients who exhibit different features and biomarkers.
- Seizure Heterogeneity: one of the factors that hinder the performance of the ES detection model is the heterogeneity of seizures. Consequently, there exists an imperative need for developing a ML model that is robust to the heterogeneity of epileptic seizure. This could be done by having a deeper understanding of seizure causes, seizure location, and how seizures spread.
- The recommendations for future work on epileptic seizure detection are as follows:
- Gain more insight into the detection of onset seizure events with real-time or near real-time monitoring of ES patients. This would enable doctors to provide timely treatment to patients before the onset of ES. Additionally, constant monitoring of ES patients using wearable EEG devices connected to smartphones and Internet of Things devices could significantly enhance the performance of machine learning models in predicting seizures.
- Develop automatic data labeling methods, as seizure detection is usually devised as a classification task that requires labeled data. EEG recordings are manually labeled by neurologists, which is a costly and time-consuming task. Thus, it is imperative to optimize the data labeling process of EEG records.

## Figures and Tables

**Table 1:** Dataset description.

Set	Symbol	Patients	Description	Segments
A	Z	Healthy	No seizure	100
B	O	Healthy	No seizure	100
C	N	Epilepsy	Inter-ictal (seizure-free)	100
D	F	Epilepsy	Inter-ictal (seizure-free)	100
E	S	Epilepsy	Seizure occurrence	100

**Table 2:** Extracted features from each coefficient.

Features	Coeff. A5	Coeff. D3	Coeff. D4	Coeff. D5
Minimum	f1	f11	f21	f31
Maximum	f2	f12	f22	f32
Median	f3	f13	f23	f33
Mean	f4	f14	f24	f34
Standard Deviation	f5	f15	f25	f35
Variance	f6	f16	f26	f36
Root Mean Square	f7	f17	f27	f37
Kurtosis	f8	f18	f28	f38
Skewness	f9	f19	f29	f39
No. of Zero Crossings	f10	f20	f30	f40

**Table 3:** Baseline results of each classifier.

Features	SVM	KNN	DT	RF
Accuracy	0.960	0.952	0.960	**0.968**
Precision	0.857	0.880	0.885	**0.889**
Recall	**0.960**	0.880	0.920	**0.960**
F1-score	0.906	0.880	0.902	**0.923**
AUC	0.960	0.952	0.960	**0.968**
Computational time (s)	1.644	**1.266**	1.297	1.978

**Table 4:** Selected features from FS-BPSO.

Features	Selected	Features	Selected	Features	Selected	Features	Selected
f1	x	f11		f21		f31	
f2		f12		f22	x	f32	
f3		f13		f23		f33	
f4	x	f14		f24		f34	
f5		f15		f25	x	f35	
f6		f16		f26	x	f36	
f7	x	f17		f27		f37	
f8		f18	x	f28		f38	
f9		f19		f29		f39	x
f10		f20		f30	x	f40	x

**Table 5:** Hyperparameters search space.

Classifier	Hyperparameter Search Space
SVM	C; float number in range (0, 50) kernel: linear, polynomial, rbf, sigmoid
KNN	K: even numbers in range (3, 20) Weight: uniform, distance Distance: Euclidean, Manhattan, Chebyshev, Minkowski
DT	max_depth: integer numbers in range (3, 50) min_samples_leaf: integer numbers in range (3, 100) min_samples_split: integer numbers in range (2, 50)
RF	max_depth: integer numbers in range (5, 50) min_samples_leaf: integer numbers in range (2, 11) min_samples_split: integer numbers in range (2, 11) n_estimator: integer numbers in range (10, 100) criterion: gini, entropy

**Table 6:** Confusion matrix for binary classification.

	Predicted Negative (0)	Predicted Positive (1)
**Actual Negative (0)**	True Negative (TN)	False Positive (FP)
**Actual Positive (1)**	False Negative (FN)	True Positive (TP)

**Table 7:** Optimal hyperparameters found by random search.

Classifier	Predicted Negative (0)	Accuracy of the Training Set from RS
SVM	C: 17.9384479998486	99.2%
	Kernel: rbf	
KNN	K: 5	98.6%
	weight: distance	
	distance: euclidean	
DT	max_depth: 37	97.3%
	min_samples_leaf: 3	
	min_sample_split: 37	
RF	max_depth: 46	98.4%
	min_samples_leaf: 2	
	min_sample_split: 4	
	n_estimator: 96	
	criterion: entropy	

**Table 8:** Initial result summary.

	SVM	KNN	DT	RF
Accuracy	**0.984**	0.976	0.968	0.968
Precision	**0.960**	0.958	0.889	0.889
Recall	**0.960**	0.920	0.960	0.960
F1-score	**0.960**	0.939	0.923	0.923
AUC	**0.980**	0.960	0.960	0.960
Computational time (s)	1.22	1.140	**1.087**	1.667

**Table 9:** Rate of change between the proposed model and baseline results.

	SVM	KNN	DT	RF
Accuracy	+2.4%	**+2.5%**	+0.8%	-
Precision	**+10.7%**	+8.1%	+0.4%	-
Recall	-	**+4.3%**	+4.2%	-
F1-score	+5.6%	**+6.3%**	+2.3%	-
AUC	**+2.0%**	+0.8%	-	−0.8%
Computational time (s)	**−46.5%**	−11.1%	−19.3%	−18.7%

**Table 10:** Computational time between baseline and proposed models.

	SVM	KNN	DT	RF
Baseline	1.644	1.266	1.297	1.978
Proposed Model	1.122	1.140	1.087	1.667
Percent reduced	−46.5%	−11.1%	−19.3%	−18.7%

**Table 11:** Results comparison with key reference for combination ABCD-E.

	SVM	KNN	DT	RF	Key Ref.
Accuracy	**98.40%**	97.60%	96.80%	06.80%	97.10%
Sensitivity	**96.00%**	92.00%	**96.00%**	**96.00%**	93.62%
Specificity	**99.00%**	**99.00%**	97.00%	97.00%	97.94%

**Table 12:** Results of SVM classifier over 10 trials.

	Accuracy	Precision	Recall	F1-Score	AUC
Trial 1	0.984	0.960	0.960	0.960	0.980
Trial 2	0.968	0.889	0.960	0.923	0.960
Trial 3	0.968	0.889	0.960	0.923	0.960
Trial 4	0.968	0.889	0.960	0.923	0.960
Trial 5	0.984	0.960	0.960	0.960	0.980
Trial 6	0.984	0.960	0.960	0.960	0.980
Trial 7	0.976	0.923	0.960	0.941	0.970
Trial 8	0.968	0.889	0.960	0.923	0.960
Trial 9	0.968	0.889	0.960	0.923	0.960
Trial 10	0.976	0.923	0.960	0.941	0.970
**Mean**	**0.974**	**0.917**	**0.96**	**0.938**	**0.968**
**Std**	**0.007**	**0.031**	**0.000**	**0.016**	**0.009**

**Table 13:** Results of KNN classifier over 10 trials.

	Accuracy	Precision	Recall	F1-Score	AUC
Trial 1	0.976	0.958	0.920	0.939	0.960
Trial 2	0.952	0.952	0.800	0.870	0.900
Trial 3	0.976	0.958	0.920	0.939	0.960
Trial 4	0.968	0.920	0.920	0.920	0.950
Trial 5	0.984	0.960	0.960	0.960	0.980
Trial 6	0.960	0.955	0.840	0.894	0.910
Trial 7	0.952	0.880	0.880	0.880	0.920
Trial 8	0.968	0.920	0.920	0.920	0.950
Trial 9	0.968	0.889	0.960	0.923	0.960
Trial 10	0.968	0.920	0.920	0.920	0.950
**Mean**	**0.967**	**0.931**	**0.90**	**0.917**	**0.944**
**Std**	**0.010**	**0.028**	**0.048**	**0.026**	**0.024**

**Table 14:** Results of decision tree classifier over 10 trials.

	Accuracy	Precision	Recall	F1-Score	AUC
Trial 1	0.968	0.889	0.960	0.923	0.960
Trial 2	0.984	1.000	0.920	0.958	0.960
Trial 3	0.952	0.852	0.920	0.885	0.940
Trial 4	0.976	0.958	0.920	0.939	0.960
Trial 5	0.984	1.000	0.920	0.958	0.960
Trial 6	0.960	0.885	0.920	0.902	0.940
Trial 7	0.944	0.821	0.920	0.868	0.940
Trial 8	0.960	0.885	0.920	0.902	0.940
Trial 9	0.960	0.885	0.920	0.902	0.940
Trial 10	0.960	0.885	0.920	0.902	0.940
**Mean**	**0.965**	**0.906**	**0.924**	**0.914**	**0.948**
**Std**	**0.012**	**0.057**	**0.012**	**0.028**	**0.010**

**Table 15:** Results of random forest classifier over 10 trials.

	Accuracy	Precision	Recall	F1-Score	AUC
Trial 1	0.968	0.889	0.960	0.923	0.960
Trial 2	0.968	0.889	0.960	0.923	0.960
Trial 3	0.960	0.885	0.920	0.902	0.940
Trial 4	0.968	0.889	0.960	0.923	0.960
Trial 5	0.976	0.923	0.960	0.941	0.970
Trial 6	0.976	0.923	0.960	0.941	0.970
Trial 7	0.968	0.889	0.960	0.923	0.960
Trial 8	0.960	0.885	0.920	0.902	0.940
Trial 9	0.968	0.889	0.960	0.923	0.960
Trial 10	0.968	0.889	0.960	0.923	0.960
**Mean**	**0.968**	**0.895**	**0.952**	**0.922**	**0.958**
**Std**	**0.005**	**0.014**	**0.016**	**0.012**	**0.010**

**Table 16:** Results of all classifiers in Scenario 1.

Classifier	Iteration	Accuracy	Precision	Recall	F1-Score	AUC
SVM	50	0.96	0.85	0.92	0.902	0.94
	100	0.96	0.885	0.92	0.902	0.94
	500	0.968	0.889	0.96	0.923	0.96
	**1000**	**0.984**	**0.96**	**0.96**	**0.96**	**0.98**
	3000	0.976	0.923	0.96	0.941	0.97
	5000	0.952	0.88	0.88	0.88	0.92
KNN	50	0.96	0.917	0.88	0.898	0.93
	100	0.968	0.92	0.92	0.92	0.95
	500	0.968	0.92	0.92	0.92	0.95
	**1000**	**0.976**	**0.958**	**0.92**	**0.939**	**0.96**
	3000	0.968	0.92	0.92	0.92	0.95
	5000	0.96	0.917	0.88	0.898	0.93
DT	50	0.984	1	0.92	0.958	0.96
	100	0.96	0.885	0.92	0.902	0.94
	500	0.976	0.958	0.92	0.939	0.96
	**1000**	**0.968**	**0.889**	**0.96**	**0.923**	**0.96**
	3000	0.96	0.885	0.92	0.902	0.94
	5000	0.968	0.92	0.92	0.92	0.95
RF	50	0.968	0.92	0.92	0.92	0.95
	100	0.968	0.885	0.92	0.902	0.94
	500	0.968	0.889	0.96	0.923	0.96
	**1000**	**0.968**	**0.889**	**0.96**	**0.923**	**0.96**
	3000	0.968	0.885	0.92	0.902	0.94
	5000	0.96	0.885	0.92	0.902	0.94

**Table 17:** Results of all classifiers in Scenario 2.

Classifier	Iteration	Accuracy	Precision	Recall	F1-Score	AUC
SVM	10	0.968	0.889	0.96	0.923	0.96
	20	0.968	0.889	0.96	0.923	0.96
	30	0.976	0.923	0.96	0.941	0.97
	**40**	**0.984**	**0.96**	**0.96**	**0.96**	**0.98**
	60	0.968	0.889	0.96	0.923	0.96
	80	0.976	0.923	0.96	0.941	0.97
	80	0.976	0.923	0.96	0.941	0.97
KNN	10	0.96	0.917	0.88	0.898	0.93
	20	0.976	0.958	0.92	0.939	0.96
	30	0.976	0.923	0.96	0.941	0.97
	**40**	**0.976**	**0.958**	**0.92**	**0.939**	**0.96**
	60	0.944	0.875	0.84	0.857	0.9
	60	0.944	0.875	0.84	0.857	0.9
	60	0.944	0.875	0.84	0.857	0.9
DT	10	0.952	0.852	0.92	0.885	0.94
	20	0.96	0.885	0.92	0.902	0.94
	30	0.96	0.885	0.92	0.902	0.94
	**40**	**0.968**	**0.889**	**0.96**	**0.923**	**0.96**
	60	0.976	0.958	0.92	0.939	0.96
	80	0.976	0.923	0.96	0.941	0.97
	100	0.968	0.862	1	0.926	0.98
RF	10	0.96	0.885	0.92	0.902	0.94
	20	0.976	0.923	0.96	0.941	0.97
	30	0.968	0.889	0.96	0.923	0.96
	**40**	**0.968**	**0.889**	**0.96**	**0.923**	**0.96**
	60	0.96	0.885	0.92	0.902	0.94
	80	0.968	0.889	0.96	0.923	0.96
	100	0.968	0.889	0.96	0.923	0.96

**Table 18:** Results comparison with recent studies.

Case	Authors	Highest Accuracy (%)
ABCD-E	A. Sharmila and P. Geethanjali [12]—Key reference	97.10
	Y. Kumar et al. [52]	97.38
	A. K. Jaiswal and H. Banka [53]	97.60
	L. Guo et al. [15]	97.77
	**Proposed approach**	**98.40**

## Data Availability

The dataset used were anonymous data obtained from http://www.meb.unibonn.de/epileptologie/science/physik/eegdata.html (accessed on 10 September 2022).

## References

[1] Sharma M., Pachori R.B., Acharya U.R. (2017). A new approach to characterize epileptic seizures using analytic time-frequency flexible wavelet transform and fractal dimension. Pattern Recognit. Lett..

[2] Pachori R.B., Patidar S. (2014). Epileptic seizure classification in EEG signals using second-order difference plot of intrinsic mode functions. Comput. Methods Programs Biomed..

[3] Rasheed K., Qayyum A., Qadir J., Sivathamboo S., Kwan P., Kuhlmann L., O’Brien T., Razi A. (2021). Machine Learning for Predicting Epileptic Seizures Using EEG Signals: A Review. IEEE Rev. Biomed. Eng..

[4] Zhou M., Tian C., Cao R., Wang B., Niu Y., Hu T., Guo H., Xiang J. (2018). Epileptic Seizure Detection Based on EEG Signals and CNN. Front. Neuroinform..

[5] Mula M., Monaco F. (2011). Ictal and Peri-Ictal Psychopathology. Behav. Neurol..

[6] Le M.T., Thanh Vo M., Mai L., Dao S.V.T. Predicting heart failure using deep neural network. Proceedings of the 2020 International Conference on Advanced Technologies for Communications (ATC).

[7] Dao S.V.T., Yu Z., Tran L.V., Phan P.N.K., Huynh T.T.M., Le T.M. (2022). An Analysis of Vocal Features for Parkinson’s Disease Classification Using Evolutionary Algorithms. Diagnostics.

[8] Si Y. (2020). Machine learning applications for electroencephalograph signals in epilepsy: A quick review. Acta Epileptol..

[9] Ahammad N., Fathima T., Joseph P. (2014). Detection of Epileptic Seizure Event and Onset Using EEG. BioMed Res. Int..

[10] Siuly, Li Y., Wen P. (2011). Clustering technique-based least square support vector machine for EEG signal classification. Comput. Methods Programs Biomed..

[11] Savadkoohi M., Oladunni T., Thompson L. (2020). A machine learning approach to epileptic seizure prediction using Electroencephalogram (EEG) Signal. Biocybern. Biomed. Eng..

[12] Sharmila A., Geethanjali P. (2016). DWT Based Detection of Epileptic Seizure From EEG Signals Using Naive Bayes and k-NN Classifiers. IEEE Access.

[13] Subasi A., Kevric J., Canbaz M.A. (2017). Epileptic seizure detection using hybrid machine learning methods. Neural Comput. Appl..

[14] Subasi A., Gursoy M.I. (2010). EEG signal classification using PCA, ICA, LDA and support vector machines. Expert Syst. Appl..

[15] Guo L., Rivero D., Dorado J., Rabuñal J.R., Pazos A. (2010). Automatic epileptic seizure detection in EEGs based on line length feature and artificial neural73 networks. J. Neurosci. Methods.

[16] Tzallas A.T., Tsipouras M.G., Fotiadis D.I. (2007). Automatic Seizure Detection Based on Time-Frequency Analysis and Artificial Neural Networks. Comput. Intell. Neurosci..

[17] Mursalin, Zhang Y., Chen Y., Chawla N.V. (2017). Automated epileptic seizure detection using improved correlation-based feature selection with random forest classifier. Neurocomputing.

[18] Sharma R.R., Varshney P., Pachori R.B., Vishvakarma S.K. (2018). Automated System for Epileptic EEG Detection Using Iterative Filtering. IEEE Sens. Lett..

[19] Wang X., Gong G., Li N., Qiu S. (2019). Detection Analysis of Epileptic EEG Using a Novel Random Forest Model Combined With Grid Search Optimization. Front. Hum. Neurosci..

[20] Yan K., Zhang D. (2015). Feature selection and analysis on correlated gas sensor data with recursive feature elimination. Sens. Actuators B Chem..

[21] Le T.M., Van Tran L., Dao S.V.T. (2021). A Feature Selection Approach for Fall Detection Using Various Machine Learning Classifiers. IEEE Access.

[22] Le M.T., Vo M.T., Pham N.T., Dao S.V. (2021). Predicting heart failure using a wrapper-based feature selection. Indones. J. Electr. Eng. Comput. Sci..

[23] El Aboudi N., Benhlima L. Review on wrapper feature selection approaches. Proceedings of the 2016 International Conference on Engineering & MIS (ICEMIS).

[24] Le T.M., Pham T.N., Dao S.V.T., Marques G., Bhoi A.K., Díez I.D., Garcia-Zapirain B. (2021). A Novel Wrapper-Based Feature Selection for Heart Failure Prediction Using an Adaptive Particle Swarm Grey Wolf Optimization. Enhanced Telemedicine and e-Health: Advanced IoT Enabled Soft Computing Framework.

[25] Pham T.N., Van Tran L., Dao S.V.T. (2021). A Multi-Restart Dynamic Harris Hawk Optimization Algorithm for the Economic Load Dispatch Problem. IEEE Access.

[26] Liu H., Yu L. (2005). Toward Integrating Feature Selection Algorithms for Classification and Clustering. IEEE Trans. Knowl. Data Eng..

[27] Chandrashekar G., Sahin F. (2014). A survey on feature selection methods. Comput. Electr. Eng..

[28] Tang J., Alelyani S., Liu H. (2014). Feature Selection for Classification: A Review. Data Classif. Algorithms Appl..

[29] Tan F. (2007). Improving Feature Selection Techniques for Machine Learning. Ph.D. Thesis.

[30] Jovic A., Brkic K., Bogunovic N. A review of feature selection methods with applications. Proceedings of the 2015 38th International Convention on Information and Communication Technology, Electronics and Microelectronics (MIPRO).

[31] Yang L., Shami A. (2020). On hyperparameter optimization of machine learning algorithms: Theory and practice. Neurocomputing.

[32] Hutter F., Kotthoff L., Vanschoren J. (2019). Automated Machine Learning: Methods, Systems, Challenges.

[33] Yu T., Zhu H. (2020). Hyper-Parameter Optimization: A Review of Algorithms and Applications. arXiv.

[34] Bergstra J., Bengio Y. (2012). Random Search for Hyper-Parameter Optimization. J. Mach. Learn. Res..

[35] Sekeroglu B., Hasan S.S., Abdullah S.M., Arai K., Kapoor S. (2020). Comparison of Machine Learning Algorithms for Classification Problems. Advances in Computer Vision.

[36] García-Gonzalo E., Fernández-Muñiz Z., Nieto P.J.G., Sánchez A.B., Fernández M.M. (2016). Hard-Rock Stability Analysis for Span Design in Entry-Type Excavations with Learning Classifiers. Materials.

[37] Kotsiantis S.B. (2007). Supervised Machine Learning: A Review of Classification Techniques. Emerg. Artif. Intell. Appl. Comput. Eng..

[38] Lan T., Hu H., Jiang C., Yang G., Zhao Z. (2020). A comparative study of decision tree, random forest, and convolutional neural network for spread-F identification. Adv. Space Res..

[39] Ali J., Khan R., Ahmad N., Maqsood I. (2012). Random Forests and Decision Trees. Int. J. Comput. Sci. Issues (IJCSI).

[40] Dimitriadis S., Liparas D., Dni A. (2018). How random is the random forest? Random forest algorithm on the service of structural imaging biomarkers for Alzheimer’s disease: From Alzheimer’s disease neuroimaging initiative (ADNI) database. Neural Regen. Res..

[41] Wolpert D.H. (1996). The Lack of A Priori Distinctions Between Learning Algorithms. Neural Comput..

[42] Andrzejak R.G., Lehnertz K., Mormann F., Rieke C., David P., Elger C.E. (2001). Indications of nonlinear deterministic and finite-dimensional structures in time series of brain electrical activity: Dependence on recording region and brain state. Phys. Rev. E.

[43] Kennedy J., Eberhart R. Particle swarm optimization. Proceedings of the ICNN’95—International Conference on Neural Networks.

[44] Mafarja M., Jarrar R., Ahmad S., Abusnaina A.A. Feature selection using binary particle swarm optimization with time varying inertia weight strategies. Proceedings of the 2nd International Conference on Future Networks and Distributed Systems.

[45] Kennedy J., Eberhart R.C. A discrete binary version of the particle swarm algorithm. Proceedings of the 1997 IEEE International Conference on Systems, Man, and Cybernetics. Computational Cybernetics and Simulation.

[46] Jahromi A.H., Taheri M. A non-parametric mixture of Gaussian naive Bayes classifiers based on local independent features. Proceedings of the 2017 Artificial Intelligence and Signal Processing Conference (AISP).

[47] Emary E., Zawbaa H.M., Hassanien A.E. (2016). Binary grey wolf optimization approaches for feature selection. Neurocomputing.

[48] Galar M., Fernandez A., Barrenechea E., Bustince H., Herrera F. (2012). A Review on Ensembles for the Class Imbalance Problem: Bagging-, Boosting-, and HybridBased Approaches. IEEE Trans. Syst. Man Cybern. Part C Appl. Rev..

[49] Vieira S.M., Mendonça L.F., Farinha G.J., Sousa J.M. (2013). Modified binary PSO for feature selection using SVM applied to mortality prediction of septic patients. Appl. Soft Comput..

[50] Mandrekar J.N. (2010). Receiver Operating Characteristic Curve in Diagnostic Test Assessment. J. Thorac. Oncol..

[51] Wang L., Xue W., Li Y., Luo M., Huang J., Cui W., Huang C. (2017). Automatic Epileptic Seizure Detection in EEG Signals Using Multi-Domain Feature Extraction and Nonlinear Analysis. Entropy.

[52] Kumar Y., Dewal M., Anand R. (2014). Epileptic seizure detection using DWT based fuzzy approximate entropy and support vector machine. Neurocomputing.

[53] Jaiswal A.K., Banka H. (2017). Epileptic seizure detection in EEG signal using machine learning techniques. Australas. Phys. Eng. Sci. Med..

